# Multi-omics analyses reveal significant differences in the gut microbiota and metabolites in children with Kawasaki disease in Northwest China

**DOI:** 10.3389/fimmu.2026.1767902

**Published:** 2026-05-11

**Authors:** Liangtao Zhao, Qi Wang, Juanjuan Chen, Jin Wang

**Affiliations:** 1 Cuiying Biomedical Research Center, The Second Hospital & Clinical Medical School, Lanzhou University, Lanzhou, China; 2 Department of Pediatric Cardiovascular, The Second Hospital & Clinical Medical School, Lanzhou University, Lanzhou, China

**Keywords:** gut microbiota, SCFA, Tryptophan metabolisms, metabolomics, Kawasaki disease, Northwest China

## Abstract

**Background:**

Kawasaki disease (KD) is a systemic vasculitis characterized by mucocutaneous lymph node syndrome and aberrant immune activation. Previous studies have indicated substantial disruptions in the gut microbiota during the acute phase of KD. However, the detailed characteristics of the gut microbiota and metabolome in children with KD, as well as their clinical relevance, remain poorly understood.

**Methods:**

31 children with KD (KDs) and age/sex-matched healthy controls (HCs) were enrolled to collect their fecal and blood samples. Shotgun metagenomic sequencing and untargeted metabolomic analyses were conducted on these samples.

**Results:**

Significant reductions in alpha diversity and microbial richness were observed in the gut microbiota of KDs at both species and genus levels. Pathogenic species including *Enterococcus avium, Streptococcus peroris* and *Clostridioides difficile* were significantly abundant in the KDs group, while beneficial species containing *Faecalibacterium prausnitzii, Anaerostipes hadrus, Akkermansia muciniphila, Eubacterium hallii, Agathobaculum butyriciproducens, Ruminococcus bicirculans*, and *Roseburia intestinalis* were markedly decreased. A total of 49 metabolic pathways were differentially enriched between the two groups, with 22 pathways including nucleotide, carbohydrate, energy, and amino acid metabolism being abundant in KDs, while the other 27 pathways were enriched in HCs. For metabolites, both fecal and blood metabolomes exhibited significant alterations. Notably, fecal metabolites including indole, L-tryptophan, L-lactic acid, 5-HETE, indol-3-acetamid, tetraethylammonium and dopaquinone were elevated in KDs, whereas butyrate, methylxanthine, phosphocholine, methylhistidine, ADP-ribose, vitamin A acid, and chenodeoxycholic acid were reduced. In plasma, cholesterol, phosphocholine, porphobilinogen, pantothenate, cortisol, bile acids and related compounds were enriched in KDs, while amino acids, indole and tryptamine derivatives, nucleotides, nucleic acids, and sugar metabolites were more abundant in HCs.

**Conclusions:**

This study represents the first systematic multi-omics investigation of KD in a pediatric population from Northwest China. It establishes a foundational resource characterizing the gut microbiome and metabolome in KD, offering novel biological insights, suggesting potential therapeutic targets, and supporting further mechanistic and clinical research.

## Introduction

Kawasaki disease (KD) is a systemic vasculitis characterized by acute fever and involvement of small- and medium-sized coronary arteries that primarily affects children under 5 years old (1), which has a high incidence in Southeast Asian countries (2). If left untreated, up to 25% of KD children may develop coronary artery (CA) aneurysms leading to coronary artery dilatation or narrowing, myocardial infarction, and ischemic cardiomyopathy (3, 4). It is reported that KD has surpassed rheumatic fever to become the leading cause of acquired heart disease among infants and children globally (5). In China, epidemiological studies from different regions show that the incidence of KD ranges from 2.34/100,000 to 54.2/100,000, with significant regional differences (6).

Although the exact triggering factors of KD remain unclear, pathological and clinical features (e.g., prolonged fever, acute onset, elevated inflammatory markers, and increased HLA-DR+ and CD4+ T cells in the intestine (7, 8)) collectively suggest that infection and immune activation might be potential contributors to KD. Gut microbiota imbalance is known to contribute to various immune and inflammatory diseases (e.g., inflammatory bowel disease, allergies, and autoimmune disorders (1, 9, 10)) by regulating the intestinal mucosal barrier, host metabolism, and immune response (11). In a longitudinal metagenomic analysis of fecal DNA from 28 patients (acute phase vs. 4–6 months post-recovery) conducted by Akiko Kinumaki et al., the authors identified significant differences (e.g., markedly increased *Streptococcus* in the acute phase (12)). In another 16S rRNA gene-based follow-up study of 26 KD patients, the authors found increased relative abundance (RA) of proinflammatory bacteria (e.g., *Ruminococcus*) and decreased anti-inflammatory microorganisms (10) Additionally, the bioactive molecules (e.g., short-chain fatty acids, bile acids, tryptophan, indole, and trimethylamine N-oxide (13)) metabolized by gut microbiota may also contribute to host immunity and inflammation. A recent preliminary study reporting reduced fecal levels of butyrate in KD patients (14). Moreover, the microbial metabolites may enter into the blood to affect host metabolism, but there is few reports of blood metabolism in KD. Luo et al. developed an LC-MS targeted metabolomics method (quantifying 276 metabolites across 60 pathways) and identified 8 differential metabolites (e.g., indole-3-propionic acid, thiamine) as potential KD diagnostic biomarkers (15). Zhu et al. identified palmitic acid as the key differential metabolite in KD patients with or without CALs, and verified it aggravates endothelial senescence via ROS, serving as a potential biomarker or therapeutic target (16). However, existing studies only focus on identify specific changes in gut microbiota composition or single detections of microbiota-related metabolites in KD. The role of specific components, metabolites, or derivatives of pathogenic microorganisms in KD pathogenesis remains unclear and research exploring the complex interactions between KD pathogenesis, gut microbiota, fecal and plasma metabolites is still limited.

Given the increasing incidence of KD in China, this study focuses on a cohort of children with KD in Northwest China. By integrating shotgun metagenomic sequencing, untargeted metabolomics, and metagenome-wide association study, we aim to characterize the changes in gut microbiota, microbial metabolites and host metabolism, as well as their correlations in KD children. Our study will not only fill gaps in regional medical data of KD in local children, but also provide potential biomarkers for treatment and prevention strategies of KD worldwide.

## Materials and Methods

### Subject enrollment and clinical assessment

The present study has been carried out to investigate the gut microbiota and metabolomic characteristics in children with KD and involved the use of human subjects, so which was conducted in accordance with the Code of Ethics of the World Medical Association (Declaration of Helsinki). The study was approved by the Medical Research Ethics Review Committee of the Second Hospital of Lanzhou University (No. 2022A–13) in 2022.

The inclusion and exclusion criteria for children with KD (**Supplementary Table 1B** Phe_KD):

1. It conforms to the clinical diagnosis of children with Kawasaki disease, and the diagnostic criteria follow the 2017 statement “Diagnosis, Treatment and Long-Term Follow-Up of Kawasaki Disease” issued by the AHA (2), including complete (cKD) and incomplete Kawasaki disease (iKD);
2. Age ≥ 1 month;
3. Within 10 days of onset (≤ 10 days, with the time of fever as the onset point), no treatment related to KD was administered;
4. Obvious fever phenomenon;
5. Treat the previous coronary artery with a z value of < 10;
6. No use of hormones or other immunosuppressants within the 1-month period prior to the onset of the disease;
7. Non-recurrent cases;
8. No definite infection, including sepsis, bacterial meningitis, acute peritonitis, bacterial pneumonia, chickenpox and influenza;
9. Without severe immune disorders such as immunodeficiency or chromosomal abnormalities;
10. No antibiotics were used within one month before the treatment;
11. Without severe immunological diseases such as immunodeficiency or chromosomal abnormalities.

### Sample Collection

According to the above inclusion and exclusion criteria, 31 children of KD and 44 healthy controls were recruited from the Department of Pediatric Cardiology of the Second Hospital of Lanzhou University from January 2022 to October 2022. Information of the research subjects, including age, gender, height, weight, residence address, mode of birth and feeding, medication use, defecation situation, family history and lifestyle, was collected through a designed questionnaire. During their hospital stay, they were subject to daily clinical follow-ups, including diet, consistency of stool and frequency of defecation, etc. In this study, all biological samples (including feces and blood) and clinical biochemical indicators were collected according to the standard operating procedures of the Second affiliated hospital of Lanzhou University in Gansu province. In details, the biological samples were immediately stored in a -80°C refrigerator after collection, and then transported quickly to the designated laboratory by dry ice for Shotgun metagenomic sequencing and untargeted metabolomic examination.

### Shotgun metagenomic sequencing

#### DNA extraction and metagenomic sequencing

DNA was extracted using the phenol/trichloromethane method after thawing the samples on ice, Extracts were then treated with DNase-free RNase and the total DNA quality and quantity were assessed through the NanoDrop Spectrophotometer ND-1000 (Thermo Fisher Scientific). The metagenomic libraries were constructed using the TruSeq DNA PCR-Free Library Preparation Kit (Illumina), and their concentrations were determined using the Qubit 2.0 fluorimeter (Invitrogen). Performing metagenomic sequencing on the BGI-Seq500 platform using paired-end libraries and a read length of 150 bases at BGI-Shenzhen (Shenzhen, China) (17).

#### Data filtering

The raw reads that had 50% low-quality bases (quality ≤ 20) or exceeding five ambiguous bases were excluded using FASTP. Aligned to the human genome (Hg19), the remaining reads were filtered in order to eliminate host DNA, using bowtie2 (-m 100-600 -v 7 -p 6 -l 30 -r 1 -M 4 -c 0.95). The remaining high-quality reads were utilized to obtain taxonomic and functional profiles.

#### Taxonomic and functional profiling

The taxonomic profiles of phyla, genera, and species were generated from the high-quality reads using Metaphlan 3.0. For functional profiling, HUMAnN 4.0 (-i input_clean_data -o output –threads 10 – memory-use maximum –remove-temp-output) was employed to efficiently profile the abundance of microbial metabolic pathways and other molecular functions from the metagenomic sequencing data according to official website and the corresponding paper (18).

#### *Alpha-* and *beta-*diversity analysis

The alpha diversity [R 4.2.1 vegan: diversity (data, index = ‘richness/Shannon/Simpson/InSimpson’)] was assessed using the richness, Shannon index, Simpson index, and Inverse Simpson index, depending on the associated taxonomic profiles. Beta diversity [(R 4.2.1 ape: pcoa (‘bray_curtis distance’, correction = “none”, rn = NULL)], R 4.2.1 vegan: diversity [data, index = ‘bray_curtis distance’)], was calculated based on the Bray-Curtis distance depending on the related taxonomic profiles. Permutational Multivariate Analysis of Variance (PERMANOVA) was performed to adjust for age in all analyses and assess the gut microbial species/genus abundance profiles through adonis function in R 4.2.1 with 1000 permutations.

#### Untargeted metabolomic examination and analysis

The untargeted metabolomic detection encompasses sample preparation and extraction, UPLC condition, and ESI-Q TRAP-MS/MS examination. The analysis of untargeted metabolomic was conducted following previously established protocols (18).

#### Differential metabolite selection between two groups

The Wilcoxon rank-sum test was used to assess differences in microbial composition and plasma metabolites between children with KD and healthy controls. The result was statistically significant with a significance level of 0.05.

#### KEGG and compound annotation and enrichment analysis

Functional analyses conducted via the KEGG Compound Database (http://www.kegg.jp/kegg/compound/) to annotate the identified microbial and host blood metabolites, and the annotated metabolites were subsequently mapped to the KEGG Pathway database (http://www.kegg.jp/kegg/pathway.html).

### Statistical analysis

Differentially elevated or depleted gut microbiota, predicted functional pathways, microbial metabolites, and host plasma metabolites were evaluated by Wilcoxon rank sum test and partial correlation test. The impact of various phenotypes on the gut microbial composition at genus and species levels, functionality, fecal and plasma metabolisms was assessed using PERMANOVA. Benjamini-Hochberg method was used to adjust the P value and control False Discovery Rate (FDR). Principle co-ordinate analysis (PCoA) was conducted using the statistical function ape comp in R (version 4.2.1) based on Bray-Curtis distances computed from the fecal and plasma metabolites profiles. The connection of significantly different gut microbes to host distinctly altered fecal/plasma metabolites was assessed using Spearman’s rank correlation.

All analyses were calculated were based on the statistical software R (Version 4.2.1). PERMANOVA: vegan, adonis (t(otu1) ~ phe [,1], data = phe, permutations = 999, na.rm = T). Wilcoxon rank sum test: wilcox. test(as.numeric(pr[i, f1]), as.numeric(pr[i, f2])). Heatmap: pheatmap(cmt,scale = “none”, cluster_row = T, cluster_col = T, display_numbers = pmt). A significance level of P < 0.05 was considered.

## Results

### Characterization of the gut microbiome structure in children with KD

A total of 31 children with Kawasaki Disease (KD) (19 males and 12 females; mean age: 2.42 ± 1.46 years) and 44 healthy controls (HCs) (20 males and 24 females; mean age: 3.92 ± 0.81 years) were enrolled in this study for fecal and blood sample collection, resulting in a total of 75 participants. No significant intergroup differences were observed in demographic and clinical characteristics, including sex, age, body mass index (BMI), birth height, birth weight, location, nationality, defecation frequency or delivery mode (**Supplementary Table 1A**). The average age of HCs was a little older than that of children with KD, but no significant difference was observed (P>0.05). However, children with KD exhibited significant abnormalities in physiological parameters (e.g., fever, bilateral conjunctival congestion, changes of lips and oral cavity, polymorphous exanthema, changes of peripheral extremities, coronary arteries with a z value of < 10), as evaluated via caregiver-reported questionnaires (**Supplementary Table 1B**). PERMANOVA (Permutational Multivariate Analysis of Variance) demonstrated significant impacts of KDs status, age, and BMI on gut microbiota composition at both the species and genus levels (**Supplementary Table 1C**).

**Figure 1:**
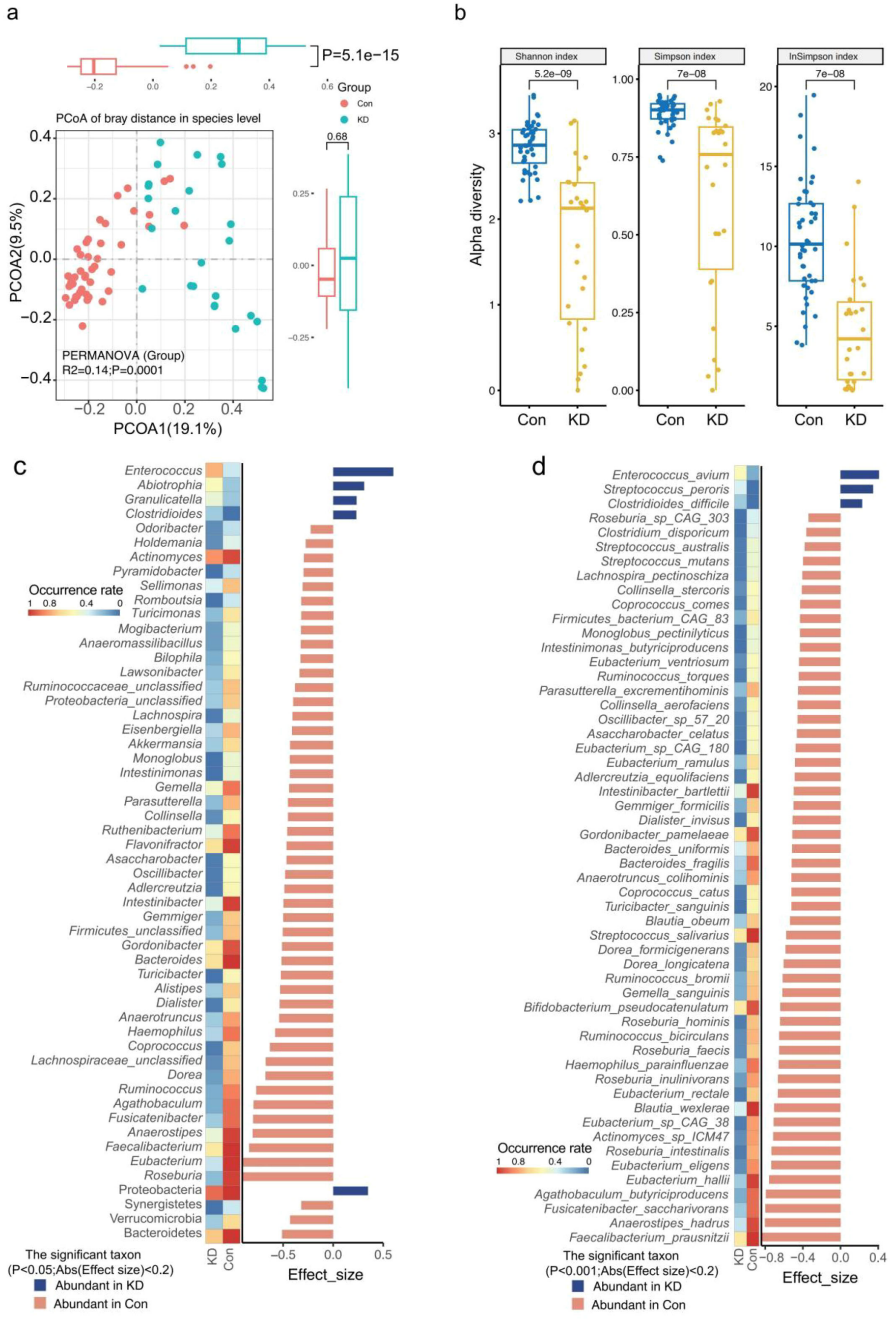
Analysis of the characteristics and differences in gut microbiota between the two groups. **(A)** PCoA based on Bray-Curtis distance at the species levels revealed significant microbial compositional differences between two groups. **(B)** Alpha diversity analysis evaluated using Shannon/Simpson/inverse Simpson indices revealed significantly lower gut microbial richness and diversity in children with KD at species levels. **(C)** Significantly different genera between two groups. **(D)** Significantly different species between two groups.

To compare changes in the gut microbiota between children with KD and HCs, metagenomic shotgun sequencing was performed. Subsequently, taxonomic profiles were identified using Metaphlan 4.0 analysis (**Supplementary Table 1E**). Principal Coordinate Analysis (PCoA) based on Bray-Curtis distance at the genera and species levels revealed significant differences in the gut microbiome composition between children with KD and HCs (species level, P = 0.001, R^2^ = 0.14; **Figure 1A**; genera level, P = 0.001, R^2^ = 0.18; **Supplementary Figure 1A**; **Supplementary Table 1C**). Alpha diversity analysis, evaluated using the Shannon, Simpson, and Inverse Simpson indices, demonstrated lower richness and diversity of both genera (**Supplementary Figure 1B**) and species (**Figure 1B**) in children with KD (**Supplementary Table 1D**).

Then, we analyzed all phyla, as well as the top 20 most abundant genera and species, between the KD and HC group (**Supplementary Tables 1F-H**). A total of nine phyla were identified; among these, the relative abundance (RA) of Proteobacteria and Verrucomicrobia was significantly higher, while that of Bacteroidetes was significantly lower in the KD group (**Supplementary Figure 1C**; **Supplementary Table 1F**). For the top 20 abundant genera, four genera including *Enterococcus, Escherichia, Veillonella* and *Lachnoclostridium* were significantly enriched in children with KD. In contrast, the RA of *Bacteroides, Faecalibacterium, Roseburia, Eubacterium, Ruminococcus, Akkermansia, Anaerostipes, Alistipes, Intestinibacter*, and *Fusicatenibacter* was significantly reduced in the KD group (**Supplementary Figure 1D**; **Supplementary Table 1G**). At the species level (top 20 abundant species), *Enterococcus faecium, Bifidobacterium longum, Bifidobacterium breve, Bifidobacterium gallicum*, and *Escherichia coli* were more prevalent in children with KD (**Supplementary Figure 1E**; **Supplementary Table 1H**). Conversely, the RA of *Faecalibacterium prausnitzii, Bacteroides vulgatus, Eubacterium rectale, Bacteroides uniformis, Ruminococcus bromii, Akkermansia muciniphila, Bacteroides fragilis, Eubacterium* sp. *CAG_180, Anaerostipes hadrus, Blautia wexlerae, Roseburia faecis*, and *Intestinibacter bartlettii* was significantly lower in the KD group (**Supplementary Figure 1E**; **Supplementary Table 1H**).

The wilcoxon rank-sum test was used for differential analysis to identify distinct microorganisms at the genus and species levels between the two groups (**Supplementary Tables 1G, H**). Children with KD exhibited significantly lower RA of multiple bacterial genera such as *Faecalibacterium, Roseburia, Eubacterium, Anaerostipes, Agathobaculum, Ruminococcus*, *Akkermansia, Bacteroides, Fusicatenibacter* all of which showed higher RA in HCs (**Figure 1C**, P<0.05). At the species level, 54 species were significantly different between the two groups (**Figure 1D**). Specifically, the RA of *Enterococcus avium, Streptococcus peroris* and *Clostridioides difficile* was significantly increased in the KD group. In contrast, 51 other bacterial species including *Faecalibacterium prausnitzii, Roseburia intestinalis, Roseburia inulinivorans, Roseburia faecis, Eubacterium hallii, Eubacterium eligens, Eubacterium sp_CAG_38, Anaerostipes hadrus, Agathobaculum butyriciproducens, Ruminococcus bicirculans, Blautia wexlerae*, *Bacteroides fragilis, Bifidobacterium pseudocatenulatum*, and *Fusicatenibacter saccharivorans* were significantly reduced in children with KD (**Figure 1D**, P<0.05).

### Functional characterization of the gut microbiota in KD

**Figure 2:**
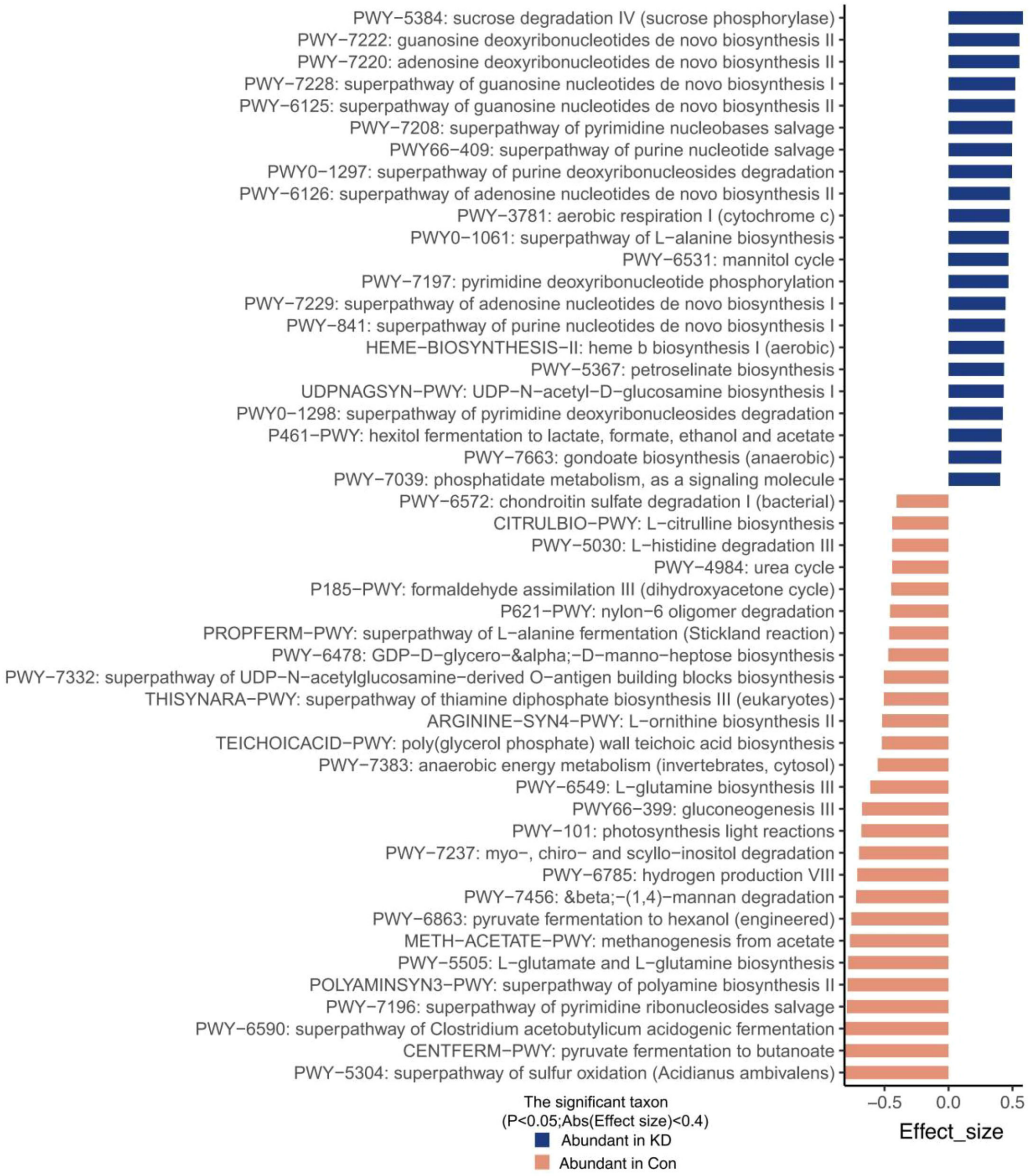
Prediction of gut microbial functional pathways. A total of 49 significantly altered predicted functional pathways were observed between the two groups, with only 22 pathways being abundant in children with KD, while the remaining 27 pathways were enriched in HCs.

HUMAnN 4.0 was utilized to accurately annotate the abundance of microbial metabolic pathways and identify specific species contributing to predictive functional profiles (**Supplementary Table 1I**). The wilcoxon rank-sum test was then used to compare pathway differences between the KD and HC group. Among the 465 total annotated metabolic pathways, 49 pathways showed significant differences between the two groups (P<0.05, **Figure 2**; **Supplementary Table 1I**).

Of these 49 pathways, 22 were significantly abundant in the KD group, categorized as follows: (1) Nucleotide metabolism: guanosine deoxyribonucleotides and adenosine deoxyribonucleotides *de novo* biosynthesis II, superpathway of guanosine nucleotides, adenosine nucleotides and purine nucleotides *de novo* biosynthesis, superpathway of pyrimidine nucleobases and purine nucleotide salvage, superpathway of purine and pyrimidine deoxyribonucleosides degradation, pyrimidine deoxyribonucleotide phosphorylation; (2) Carbohydrate metabolism: sucrose degradation IV, mannitol cycle, UDP-N-acetyl-D-glucosamine biosynthesis I, hexitol fermentation to lactate, formate, ethanol and acetate; (3) Energy and amino acid metabolism: aerobic respiration I, superpathway of L-alanine biosynthesis, phosphatidate metabolism, as a signaling molecule; (4) other pathways: heme biosynthesis I, petroselinate biosynthesis, gondoate biosynthesis (**Figure 2**; **Supplementary Table 1I**).

The remaining 27 pathways were significantly enriched in the HC group but notably reduced in the KD group, including pyruvate fermentation to butanoate, superpathway of *Clostridium acetobutylicum* metabolism, pyruvate fermentation to hexanol, superpathway of polyamine biosynthesis II, gluconeogenesis III, superpathway of L-alanine fermentation. These results indicated potential dysfunction of the gut microbiota in children with KD, although beyond the observed reductions in microbial diversity and richness, and imply more substantial changes in metabolites in the KD group (**Figure 2**; **Supplementary Table 1I**).

Interestingly, functional pathways associated with *Enterococcus avium* (a species with significantly higher RA in the KD group) were also enriched in the KD group (**Supplementary Figure 3**). Conversely, *Faecalibacterium prausnitzii* (significantly enriched in HC group) participated in pathways that were more abundant in the HC group (**Supplementary Figure 2**). These findings further confirm the consistency between species-specific alterations and functional pathway changes.

### Differential analysis of gut microbial metabolites in KD

**Figure 3:**
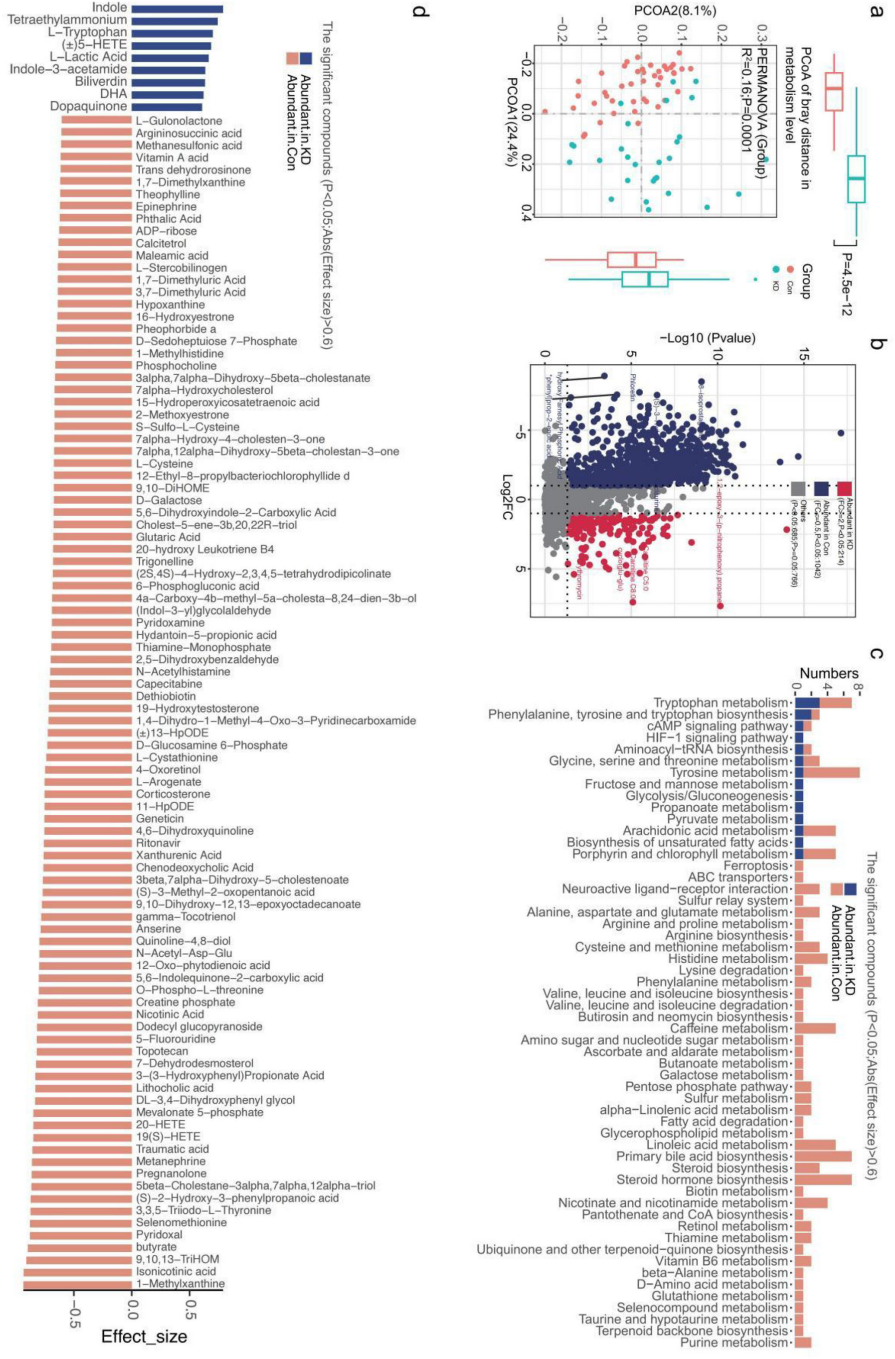
Fecal metabolome profile analysis between two groups. **(A)** PCoA based on Bray-curtis distance of fecal metabolites showed significant distinction between two groups. **(B)** Volcano plot analysis: Markedly different fecal metabolites (red: up-regulated; blue: down-regulated). **(C)** KEGG pathways showing significant correlations with varying fecal metabolites. **(D)** The abundance of significantly different fecal metabolites between two groups.

To verify alterations in gut microbial metabolites in children with KD, non-targeted metabolomics analysis was performed (**Supplementary Table 1J**). PCoA based on Bray-Curtis distance revealed significant differences in gut microbial metabolite profiles between the KD and HC group (R^2^ = 0.16, P = 0.0001) (**Figure 3A**).

A total of 2690 metabolites were detected across all subjects, of which 1256 showed significant differences in frequency between the two groups (P<0.05, **Figure 3B**; **Supplementary Tables 1J, K**). Among these, 105 fecal metabolites had a fold change (FC) >2 or <1/2 (**Figure 3B**). Specifically, nine metabolites including indol, tetraethylammoniium, L-tryptopha, 5-HETE, L-lactic acid, indole-3-acetamid were significantly enriched in the KD group. In contrast, 96 metabolites including butyrate, methylxanthine, phosphocholine, methylhistidine, ADP-ribose, L-arogenate, corticosterone 20-hydroxy leukotriene B4, 19 (S)-HETE, and N-acetylhistamine were significantly more abundant in HCs but reduced in the KD group (**Figure 3D**).

To further explore the functional relevance of these metabolites, we annotated them using the Kyoto Encyclopedia of Genes and Genomes (KEGG) database. Among the functional annotation results, metabolites enriched in the KD group were primarily associated with the following KEGG pathways: tryptophan metabolism, phenylalanine/tyrosine/tryptophan biosynthesis, arachidonic acid metabolism, HIF-1 signaling pathway, fructose and mannose metabolism, glycolysis/Gluconeogenesis, cAMP signaling pathway, propanoate metabolism, pyruvate metabolism, biosynthesis of unsaturated fatty acids. In contrast, metabolites abundant in HCs were mainly involved in neuroactive ligand-receptor interaction, ABC transporters, alanine/aspartate/glutamate metabolism, butanoate metabolism, amino sugar/nucleotide sugar metabolism, α-linolenic acid metabolism, fatty acid degradation, glycerophospholipid metabolism, and purine metabolism (**Figure 3C**).

**Figure 4:**
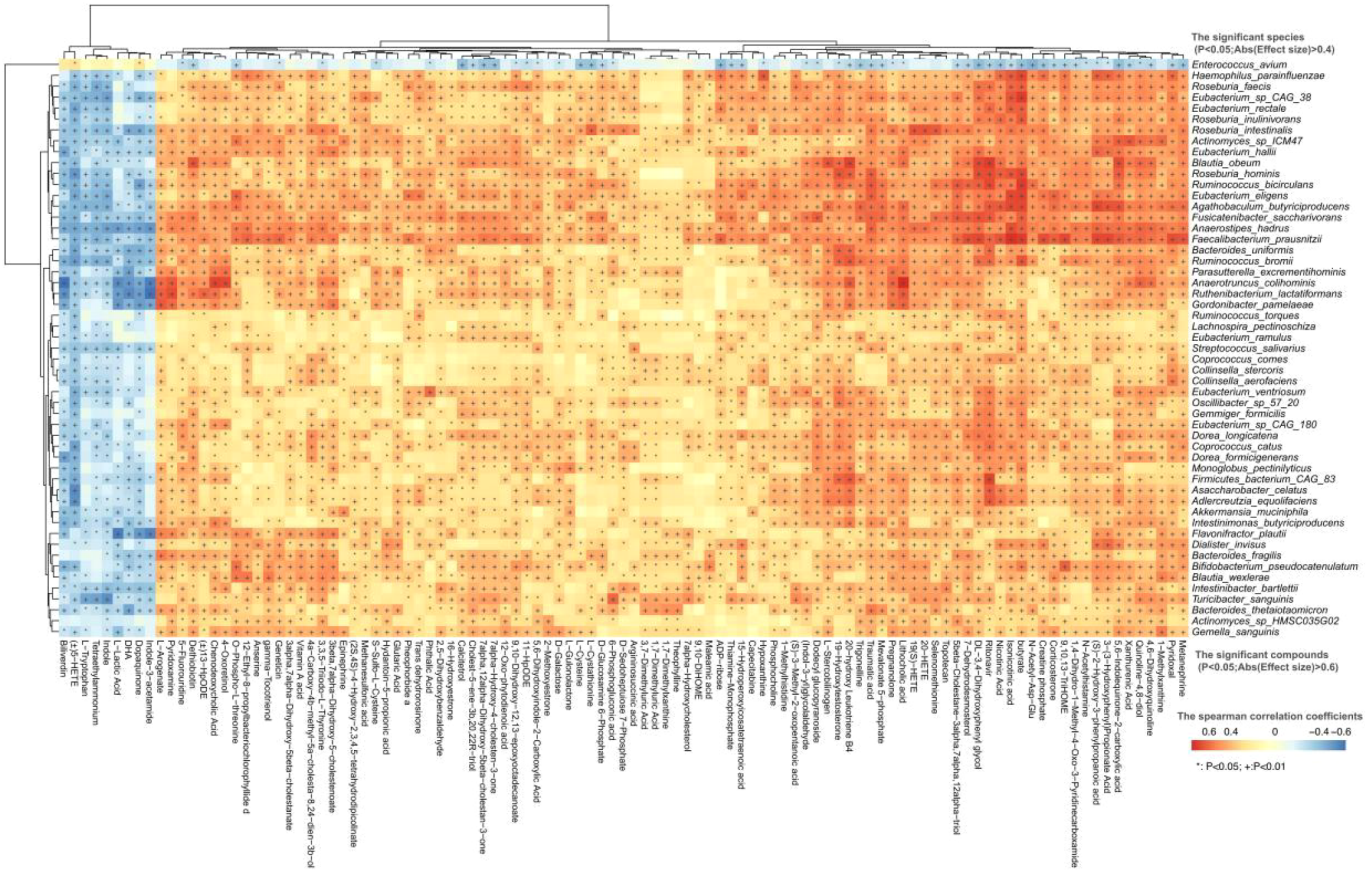
Spearman’s rank correlation was used to reveal associations between the significantly differential microbial species and the significantly changed fecal metabolites between the two groups. *P < 0.05; + P < 0.01.

To elucidate the relationship between gut microbiota and their metabolic functions in children with KD, we analyzed correlations between 53 significantly different microbial species and 105 distinct fecal metabolites. *Enterococcus avium* showed a significant positive correlation with metabolites highly abundant in the KD group, including indole, L-tryptophan, L-lactic acid, 5-HETE, indol-3-acetamid, tetraethylammonium and dopaquinone (**Figure 4**). Conversely, it exhibited a negative association with metabolites predominantly enriched in HC group (**Figure 4**).

### Differential analysis of plasma metabolites in KD

**Figure 5:**
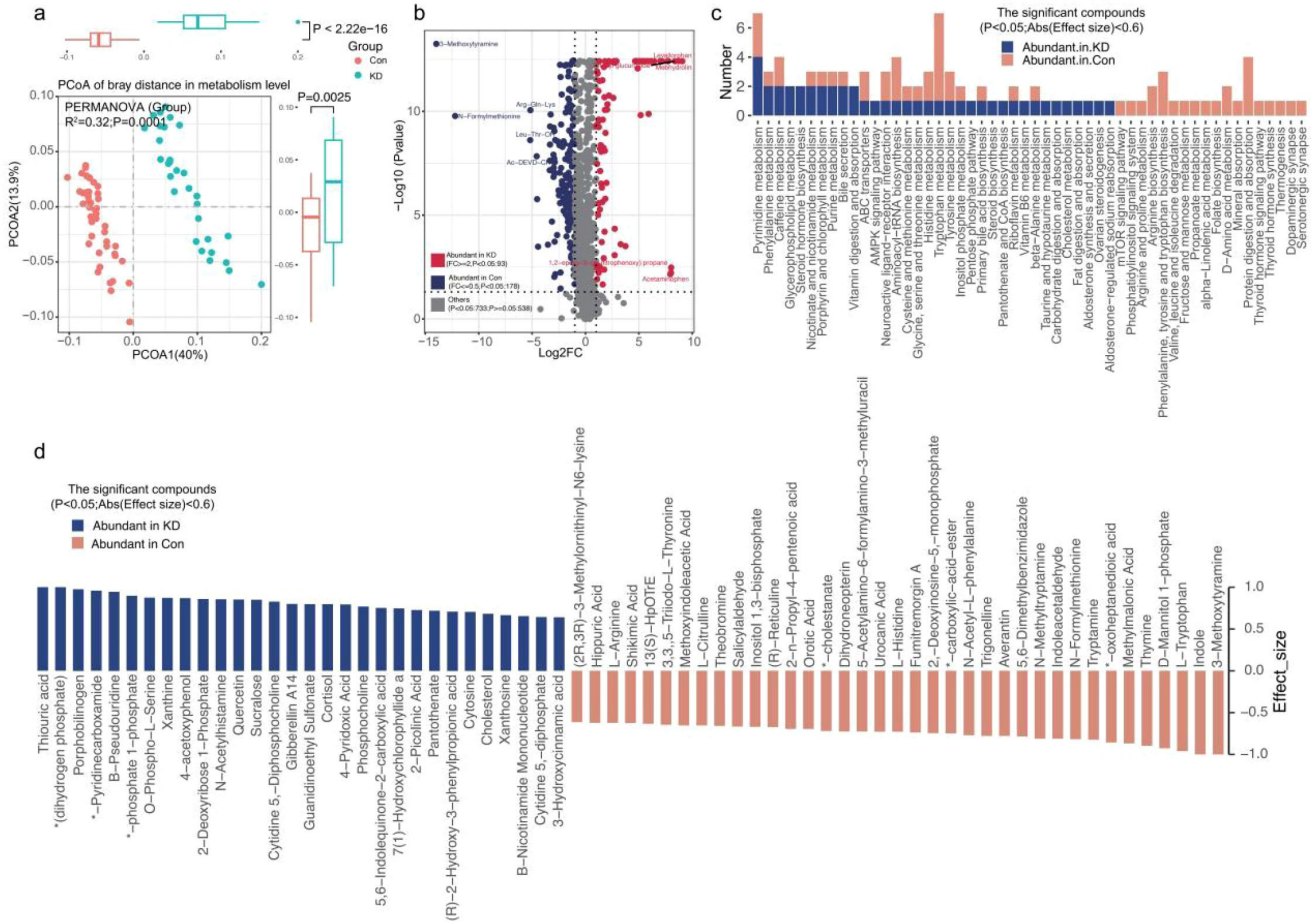
The analysis of plasma metabolome profile between two groups. **(A)** PCoA was conducted on plasma metabolites with Bray-Curtis distance and indicated significant separation of the two groups. **(B)** Volcano plot analysis identified significantly different plasma metabolites, highlighting those that were notably up-regulated (red) and down-regulated (blue). **(C)** Plasma metabolite-specific KEGG pathways with significant associations. **(D)** Heatmap analysis revealed the abundance of plasma metabolites with significant differences between the two groups.

Gut microbial metabolites can be absorbed into blood and subsequently modulate the host’s physiological state. To investigate this, we detected plasma metabolites in children with KD and HC group (**Supplementary Table 1L**). PCoA based on Bray-Curtis distance revealed a statistically significant difference in plasma metabolite profiles between the two groups (P = 0.001, R^2^ = 0.32, **Figure 5A**).

A total of 1004 plasma metabolites were detected, among which 271 showed significant differences between the KD and HC groups (P<0.05, **Figure 5B**). Specifically, 93 metabolites were more abundant in the KD group, while 178 were enriched in HC group (**Supplementary Table 1l**). KEGG pathway annotation revealed that metabolites enriched in HCs were primarily associated with the following pathways: mTOR signaling pathways, phosphatidylinositol signaling system, arginine/proline metabolism, arginine biosynthesis, phenylalanine/tyrosine/tryptophan biosynthesis, valine/leucine/isoleucine degradation, fructose/mannose metabolism, propionate metabolism, alpha-linolenic acid metabolism, folic acid biosynthesis, D-amino acid metabolism, mineral absorption, protein digestion and absorption, thyroid Adenine signaling pathways, thyroid hormone synthesis, heat production, dopaminergic synapses, etc. (**Figure 5C**). In contrast, metabolites enriched in the KD group were primarily involved in glycerophospholipid metabolism, steroid hormone biosynthesis, vitamin digestion and absorption, AMPK signaling pathway, pentose phosphate pathway, steroid biosynthesis, biotin and coenzyme A biosynthesis, taurine and hypotaurine metabolism, carbohydrate digestion and absorption, cholesterol metabolism, fat digestion and absorption, aldosterone synthesis and secretion, ovarian steroidogenesis, and aldosterone-regulated sodium reabsorption (**Figure 5C**). Additionally, 30 and 37 significant metabolites in the KD and HC groups, respectively, were annotated to corresponding metabolic pathways (**Figure 5D**; **Supplementary Table 1M**).

We further analyzed correlations between gut microbiota and plasma metabolites (**Supplementary Figure 4**). Microbial species with significant differences in RA between the KD and HC groups, including *Enterococcus avium, Faecalibacterium prausnitzii, Anaerostipes hadrus, Agathobaculum butyriciproducens, Blautia wexlerae*, *Eubacterium hallii, Eubacterium eligens, Eubacterium sp_CAG_38, Roseburia intestinalis, Ruminococcus bicirculans*, and *Bifidobacterium pseudocatenulatum*, exhibited obvious associations with plasma metabolites. Notably, *Enterococcus avium* (enriched in KD) showed a significant positive correlation with plasma metabolites abundant in the KD group such as porphobilinogen, thiouric acid, phosphocholine, cortisol, N-acetylhistamine, 5-diphosphocholine and cholesterol. In contrast, it displayed a negative correlation with metabolites highly abundant in HC group, including indole, L-tryptophan, D-mannitol, N-formylmethionine, tryptamine, indoleacetaldehyde (**Supplementary Figure 4**). These plasma metabolites are involved in various physiological processes, including the regulation and differentiation of immune cells, maintenance of immune homeostasis, and modulation of inflammation-related signaling pathways (**Supplementary Figure 4**).

## Discussion

Recent studies have identified significant gut microbiota disruptions in Kawasaki disease (KD) patients; however, previous research has not systematically elucidated the relationship between dysregulated microbial species and associated metabolic pathways, microbial metabolites, and plasma metabolites, nor their potential roles in KD pathogenesis. To address this gap, our study utilized shotgun metagenomic sequencing and untargeted metabolomics to analyze fecal and blood samples from children with KD and age-/sex-matched healthy controls (HCs) in northwest China. The goal was to characterize the gut microbiota, fecal metabolome and plasma metabolome in KD. Our findings revealed markedly reduced gut microbiota diversity and richness in KD children, alongside significant differences in fecal and plasma metabolite compositions between KD and HC groups.

Gut microbiota dysbiosis and increased intestinal permeability—key drivers of intestinal barrier dysfunction—are widely recognized as pathogenic factors in various inflammatory and metabolic diseases, including cardiovascular diseases (CVDs) (8). Consistent with this, our study found that KD children exhibited significantly lower gut microbiota diversity and richness compared to HCs. At both the genus and species levels, α-diversity and β-diversity differed significantly between the two groups, aligning with existing research (12). Notably, we identified a significant increase in the relative abundance (RA) of previously unreported “unfriendly” bacterial species in KD patients, including *Enterococcus avium*, *Streptococcus peroris*, and *Clostridioides difficile*. *Enterococcus avium* is an opportunistic pathogen, it proliferate excessively during gut microecological dysbiosis (19) and accounts for 5-15% of adult endocarditis cases (20). Its growth may stimulate the immune system to produce pro-inflammatory cytokines (e.g., tumor necrosis factor [TNF], interleukin-6 [IL-6]), promoting translocation of microbiota and their metabolites into the bloodstream, activating systemic immune responses, and exacerbating inflammation (21). Additionally, a prior study linked increased *Enterococcus faecalis* abundance in children with KD to antibiotic resistance genes (ARGs), suggesting a potential role of the *Enterococcus* genus in intravenous immunoglobulin (IVIG) therapy resistance (22). *Streptococcus peroris* has been identified in numerous studies as being associated with the development of cardiovascular inflammation (23). It can enter the bloodstream through oral ulcers or gingival bleeding resulting from periodontal disease, adhere to damaged cardiac endothelium, and trigger infective endocarditis (24). Bacterial components like lipopolysaccharides (LPS) can activate macrophages to release TNF-α and IL-6, which drive vascular endothelial inflammation and atherosclerosis (25). Meta-analyses show that periodontal disease increases CVD risk by 19-24% (26), underscoring a potential link between *S. peroris* and KD-related cardiovascular pathology. Research on KD and *C. difficile* is limited, but Mahnic et al. found that *C. difficile* proliferates in infants with gut microbiota dysbiosis (characterized by reduced α-diversity and increased *Escherichia coli*) (27). Additionally, children with inflammatory bowel disease (IBD) are particularly susceptible to *C. difficile* infection (28). In our study, KD children exhibited increased *Escherichia coli* abundance and significant differences in diarrhea severity and frequency compared to HCs (P = 0.0003)—including higher diarrhea incidence, increased defecation frequency, and altered stool consistency. Therefore, we infer that *C. difficile* abundance may correlate with diarrhea severity in KD. Collectively, these findings suggest that gut microbiota dysbiosis-induced increases in detrimental bacterial species and their metabolites may disrupt immune homeostasis and indirectly drive cardiovascular inflammation in KD.

Cardiovascular diseases (CVDs) are typically associated with reduced SCFA-producing bacteria, increased opportunistic pathogens, and systemic inflammation (1). Our study found that KD children showed a significantly reduced gut microbiota diversity, which may stem from the depletion of highly abundant beneficial bacterial species, including *Faecalibacterium prausnitzii, Roseburia intestinalis, Roseburia inulinivorans, Roseburia faecis, Eubacterium hallii, Eubacterium eligens, Eubacterium sp_CAG_38, Anaerostipes hadrus, Agathobaculum butyriciproducens, Ruminococcus bicirculans, Blautia wexlerae*, *Bacteroides fragilis*, and *Fusicatenibacter saccharivorans*, most of which are major producers of the SCFA butyrate. This observation aligns with prior CVD-related research: Hypertensive patients show depleted butyrate-producing bacteria (e.g. *Roseburia, Faecalibacterium, Coprococcus*, and *Ruminococcaceae*) (29, 30). Coronary artery disease patients exhibit reduced abundance of butyrate producers (*Faecalibacterium, Roseburia*, and *Eubacterium rectale*) and increased opportunistic pathogens including *Escherichia-Shigella, Ruminococcus gnavus*, and *Enterococcus* (31). Atherosclerosis patients have lower levels of butyrate-producing *Roseburia* and *E. rectale* (32). Butyrate can stimulate villus growth to produce mucin to protect the intestinal mucosa, activates AMPK to promote tight junctions to prevent intestinal content leakage into the bloodstream, and inhibits NF-κB activation while upregulating PPAR-γ and suppressing IFN-γ to reduce intestinal mucosal inflammation (33). Supplementation with SCFAs alone or in combination lowers blood pressure in hypertensive mice (34) and reduces atherosclerosis incidence (35). Colonization of atherosclerotic mice with *R. intestinalis* (a butyrate-producer) enhances intestinal barrier function, inhibiting inflammation and reducing atherosclerosis and endotoxemia (36). Additionally, *R. intestinalis* and its metabolite butyrate can significantly reduce abdominal aortic aneurysms development in mice by suppressing inflammation, NOX2-dependent neutrophil extracellular trap formation, neutrophil infiltration, and abnormal vascular smooth muscle cell phenotypic transformation (37).

KD, an immune-related disorder, arises from interactions between genetic/environmental susceptibility factors and infectious triggers, followed by acute-phase abnormal immune responses (14). Butyrate can regulate Th17/Treg differentiation (38, 39), and reduced butyrate in KD may disrupt this balance—contributing to excessive cytokine production and KD development (14). Building on this, our study further identifies and validates additional depleted butyrate-producing bacteria in KD, confirming that gut microbiota dysbiosis reduces SCFA (especially butyrate) levels. This metabolic deficiency may elevate inflammatory cytokines and chemokines, disrupt Th17/Treg balance, and induce aberrant immune responses in KD.

The fecal metabolome is a reliable indicator of microbial activity and an indirect reflection of fecal microbiota composition. Beyond gut microbiota changes, our untargeted metabolomics analysis revealed significant differences between KD and HC groups. The KD patients had significantly higher levels of differential metabolites, including indole, L-tryptophan, 5-HETE, L-lactic acid, indole-3-acetamide, and dopamine. Meanwhile, KD patients had significantly lower fecal butyrate levels—consistent with the depletion of butyrate-producing bacteria observed earlier. These results indicated reduced degradation of the aforementioned metabolites in KD, and their levels positively correlated with the abundance of *Enterococcus avium*—a key enriched bacterium in KDs. L-tryptophan (Trp), an essential aromatic amino acid, is a precursor for host and microbial metabolites and plays a critical role in immune regulation (40).

Gut Trp is mainly metabolized into indole and its derivatives (e.g., indole-3-aldehyde (IAld), indole-3-acid-acetic (IAA), indole-3-propionic acid (IPA), indole-3-acetaldehyde (IAAld)), which act as aryl hydrocarbon receptor (AhR) ligands to activate AhR in intestinal epithelial cells (41). AhR signaling is essential for maintaining intestinal homeostasis and local immune responses (42), which showed reduced gut AhR expression in IBD patients (43). In addition, the serum level of indolepropionic acid (IPA) is reduced in patients with ulcerative colitis (UC) (44). Studies on some mouse models of IBD have also demonstrated that AhR deficiency exacerbates experimental colitis induced by T-cell transfer or dextran sulfate sodium (DSS) (43, 44). Therefore, reduced fecal Trp metabolism in KD may lower AhR ligand levels, impairing AhR signaling and inducing intestinal inflammation. While indole (a major AhR activator) protects intestinal epithelial structure/function under normal conditions (45), excessive indole can exacerbate intestinal inflammation: in a mouse colitis model, indole-mediated overactivation of the intestinal 5-HT receptor HTR2B amplified inflammatory responses (46). Furthermore, indole sulfate (IS), a hepatic metabolite of indole, accumulates in patients with chronic kidney disease (CKD), damaging vascular endothelium, promoting inflammation, and accelerating atherosclerosis (47). 5-HETE, a arachidonic acid metabolite, promotes immune cell recruitment and activation, amplifies inflammatory mediator networks, and modulates inflammation-associated signaling pathways. It is a product of 5-LOX (highly expressed in the intestinal mucosa of patients with IBD) and contributes to inflammatory cell activation and increased vascular permeability (48, 49). Additionally, 5-HETE contributes to macrophages foam cell formation in atherosclerotic plaques, accelerating plaque instability (50). Together, these findings suggest that gut microbiota dysbiosis in KD exacerbates inflammation not only by reducing protective SCFAs (e.g., butyrate) (and triggering cytokine storms) but also by altering metabolite levels to disrupt host physiological functions.

The fluctuations in serum and endogenous metabolites reflect the metabolic and immune status of peripheral organs such as the heart, liver, and muscles (51). Our untargeted plasma metabolomics analysis identified 30 significantly increased metabolites in the serum of KD patients, including cortisol, xanthine, quercetin, porphobilinogen, 5,6-indolequinone-2-carboxylic acid, and cholesterol, which are all positively associated with inflammatory responses. As a stress hormone, excessive elevation of cortisol may disrupt immune homeostasis, exacerbates tissue damage, and enhances chronic inflammation (52). Increased levels of xanthine in plasma may result from insufficient xanthine oxidoreductase (XOR) activation. Reduced XOR/xanthine oxidase (XO) activity increases the production of reactive oxygen species (ROS), causing oxidative stress-induced damage to vascular endothelium and cardiovascular tissues (53). Additionally, 5,6-Indoloquinone-2-carboxylic acid, as a oxidative metabolite of tryptophan metabolism, can damage immune cells and promote inflammation via NLRP3 inflammation activation (54).

We further explored gut microbiota-plasma metabolome correlations and found that these pro-inflammatory metabolites in KD were most strongly positively correlated with *E. avium*. In contrast, significantly reduced serum metabolites in KD (e.g., Trp, indole, butyrate) were negatively correlated with *E. avium*. These observations align with prior research. Decreased serum Trp was reported to impair the activity and function of immune cell, weakening host defenses and increasing susceptibility to infections (55). In another study related to distinct chronic inflammatory diseases (CIDs), serum Trp was found to be negatively correlated with C-reactive protein (CRP) in most patients, increased Trp degradation is a common CID metabolic feature that impairs systemic immunity (56). Additionally, another study involving 362 healthy adults found that the ratio of kynurenine-to-tryptophan (Kyn/Trp) was significantly positively correlated with inflammatory markers (neopterin, IP-10, TNF-α, CRP, and IL-10), linking Trp metabolism to systemic immune activation (57). Consistent with these findings, altered Trp levels in both fecal and blood samples of KD patients further confirm inflammatory activation. Additional studies shows that decreased serum indole can inhibit intestinal epithelial adherens junction protein expression (58), reduce the secretion of mucin MUC2 (59), suppress Treg differentiation and activates NLRP3 inflammasomes (60) to lead to intestinal barrier dysfunction, inflammasome activation, reduced phagocytic function, and systemic low-grade inflammation.

Similarly, reduced serum butyrate loosens epithelial cell connections, decreases tight junction proteins, and increases intestinal permeability, which allowing bacterial endotoxins to enter the bloodstream and trigger low-grade inflammation (61). It also overactivates the NF-κB pathway or NLRP3 inflammasome, intensifying inflammation and further damaging the intestinal barrier (62).

These serum metabolite changes not only confirm inflammatory activation in KD but also highlight the role of gut microbiota-plasma metabolite interactions in KD onset and progression.

## Conclusions

In summary, our study characterized the composition and function of the gut microbiota and identified differences in fecal and plasma metabolites between children with KD and HCs using multi-omics data. Our findings suggest that KD onset is closely associated with significant changes in the gut microbiota metabolic profile and systemic metabolic environment, characterized by depletion of beneficial symbiotic bacteria with anti-inflammatory or immunomodulatory properties; Overgrowth of pro-inflammatory or opportunistic pathogenic bacteria; educed circulating levels of protective metabolites; and Increased concentrations of pro-inflammatory or cytotoxic metabolites. These disruptions in gut and systemic metabolic balance may act synergistically to drive the onset and development of KD.

## Data Availability

The datasets presented in this study can be found in online repositories. The names of the repository/repositories and accession number(s) can be found in the article/**Supplementary Material**.
