# Supplementary material for: Multi-omics analyses reveal significant differences in the gut microbiota and metabolites in children with Kawasaki disease in Northwest China
Source: Front Immunol. 2026 May 11;17:1767902. doi: 10.3389/fimmu.2026.1767902 (PMC13199117; doi:10.3389/fimmu.2026.1767902)
Supplement: Supplementary file 1 [file DataSheet1.docx]

**Supplementary material**

The present supplementary materials contain **four supplementary figures** and **one Supplementary Table.**

**Table S1** comprises 13 sheets, encompassing comprehensive data on taxonomic and functional profiling, metabolomic data, as well as detailed analyses corresponding to all figures. All supplementary figures have been consolidated into a single Word document.However, given its substantial size, we intend to keep Table S1 as separate individual sheets. Descriptions and headers for each sheet within Table S1 are included in the appendix of this manuscript. Please review.

# Supplementary Figures

**
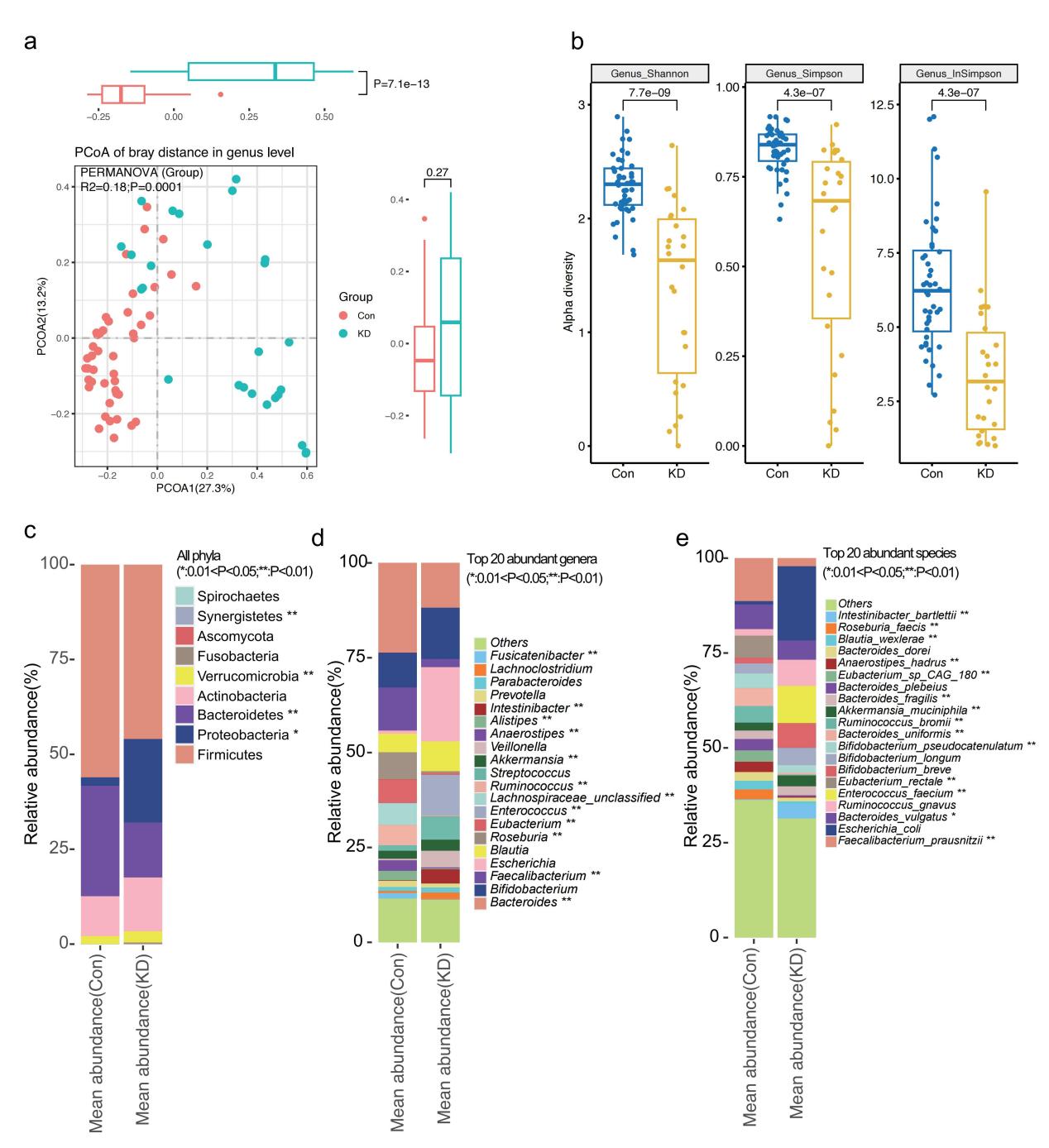
**

**Figure S1** All phyla, top 20 most abundant genera and species in the gut microbiota of children with KD and healthy controls.

(a) PCoA based on Bray-curtis distance at the genera levels revealed significant differences between two groups. (b) Alpha diversity analysis evaluated using Shannon/Simpson/inverse Simpson indices revealed significantly lower diversity in children with KD at genus. (c) All phyla distribution in the two groups. (d) Top 20 abundant genera in the two groups. (e) Top 20 abundant species in the two groups. *P < 0.05. The table in the figure presents the exact P-values and enrichment directions of taxa with significant differences.

**
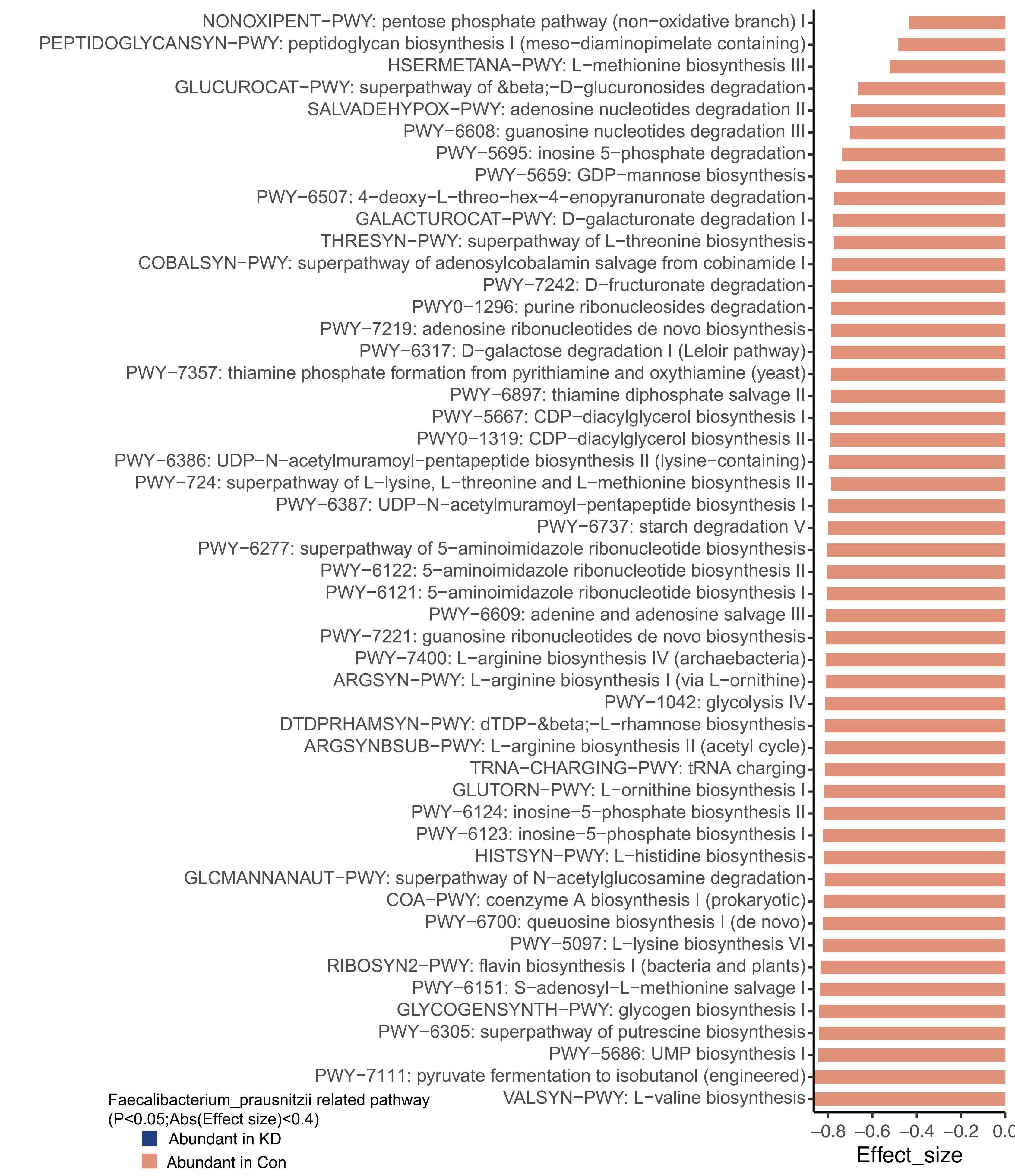
**

**Figure S2** Significantly different pathways participated by *Faecalibacterium prausnitzii.*

**
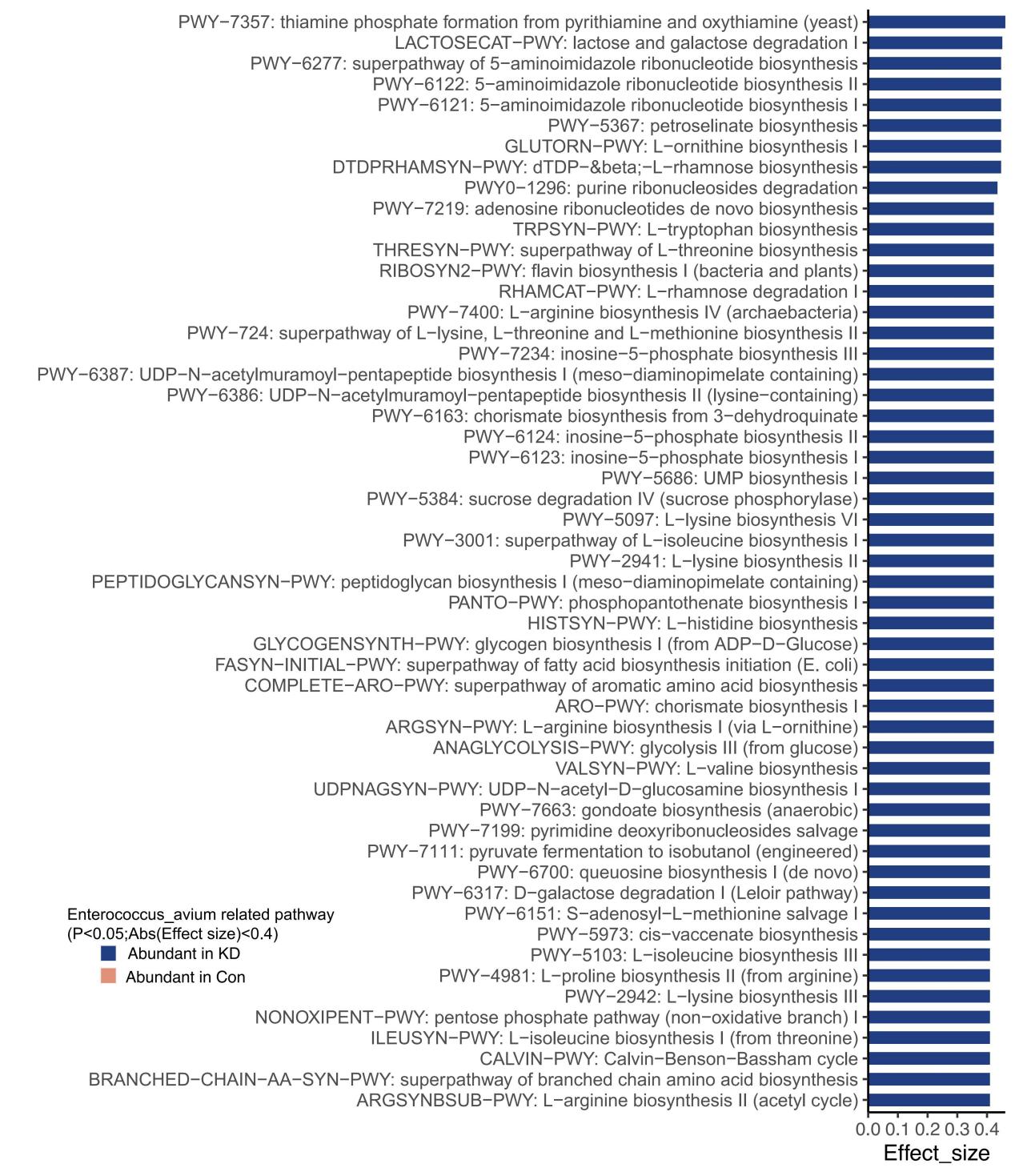
**

**Figure S3** Significantly different pathways participated by *Enterococcus avium.*

**
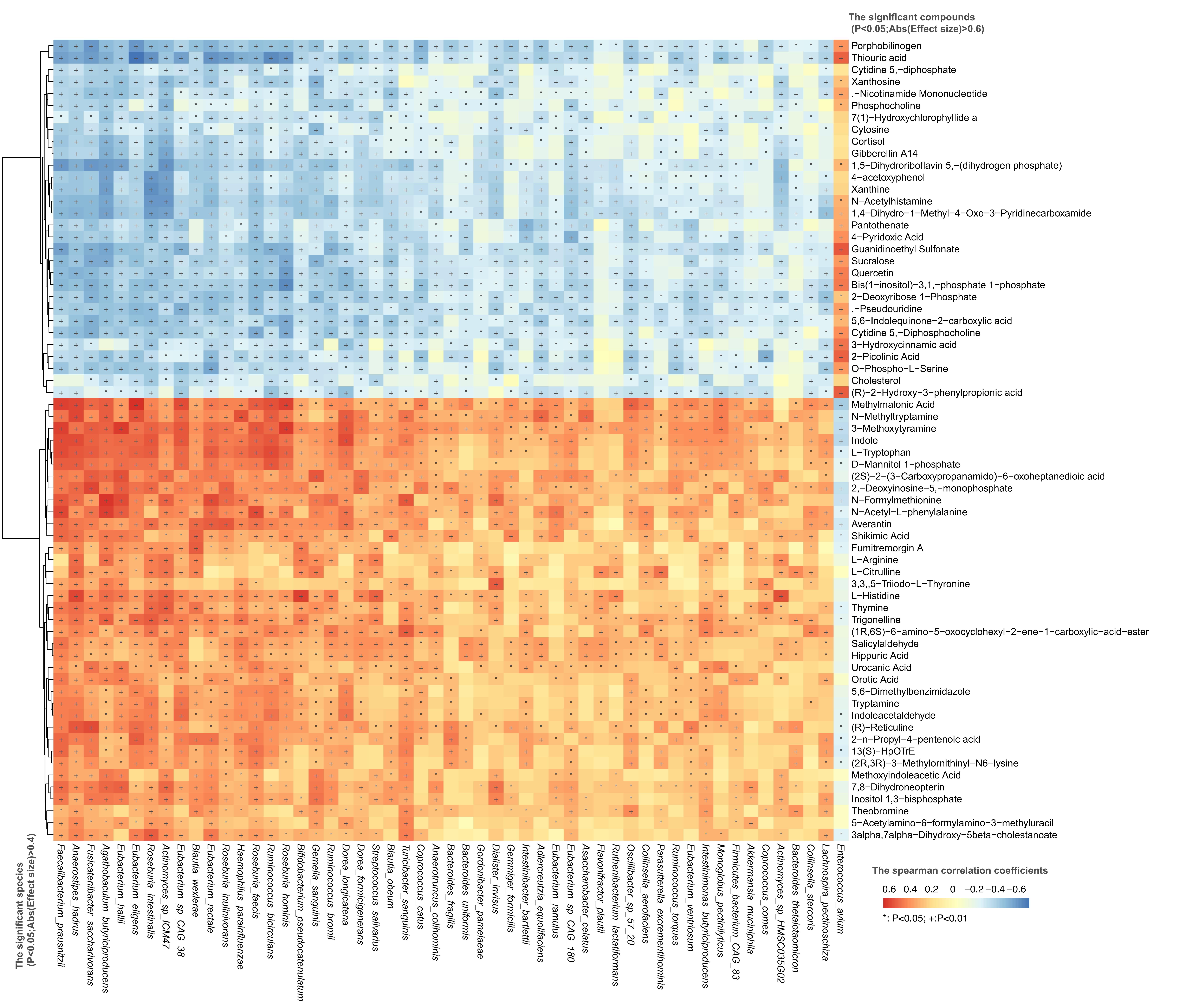
**

**Figure S4** Spearman’s rank correlation was used to reveal associations between the significantly differential microbial species and plasma metabolites between the two groups. * P < 0.05; + P < 0.01.

**Table S1**

**Table S1**A. Phenotypes of the subjects.

**Table S1B**. Phenotypes of the children with KD.

**Table S1C**. PERMANOVA of various phenotypes on gut microbial composition.

**Table S1D**. Diversity calculation of the gut microbiota at phyla, genera, and species levels.

**Table S1E**. Taxonomic profiling of the gut microbiota.

**Table S1F**. Phyla analysis.

**Table S1G**.Genera analysis.

**Table S1H**. Species analysis.

**Table S1I**. Functional analysis of the gut microbiota.

**Table S1J**. Fecal metabolites analysis of the gut microbiota.

**Table S1K**. KEGG_map analysis of Fecal metabolites.

**Table S1L**. Plasma metabolites analysis of the gut microbiota.

**Table S1M**. KEGG_map analysis of plasma metabolites.
